# Supplementary material for: Modular quantum computation in a trapped ion system
Source: Nat Commun. 2019 Oct 16;10:4692. doi: 10.1038/s41467-019-12643-2 (PMC6795904; doi:10.1038/s41467-019-12643-2)
Supplement: Supplementary file 1 — Supplementary Information [file 41467_2019_12643_MOESM1_ESM.pdf]

Supplementary Information for:

Modular Quantum Computation in a Trapped Ion System

Kuan et al.

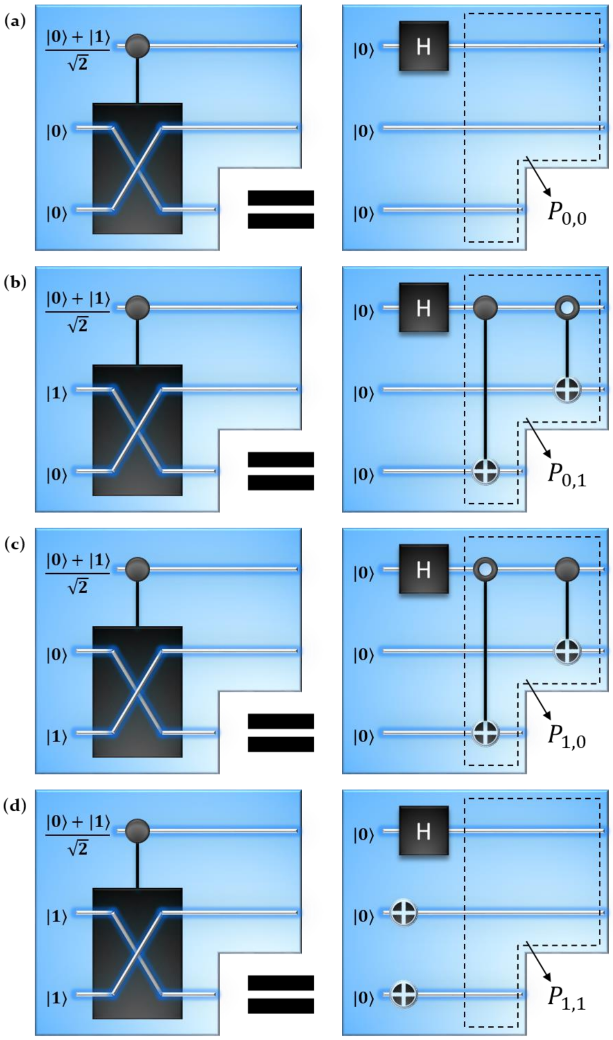

Supplementary Figure 1. **Circuits of implementing pre-processing.** Pre-processing involves simulating the desired mixed state through a statistical mixture of four separate circuits, where the output of each circuit is the same as the corresponding circuit in left column.

SUPPLEMENTARY NOTE 1: EXPERIMENTAL DETAILS

Our experiment consists of three logical qubits: The control qubit C that is encoded within the hyperfine levels of an $^{171}\mathrm{Yb}^+$ ion and the reservoir qubits X and Y that correspond the ground and first excited states of the ion's two motional degrees of freedom. Operations on C are enabled by microwave pulses, which inact the Hamiltonians

$$R_0(\chi,\phi) = \exp\left[-\frac{i\chi}{2}\left(e^{-i\phi}\left|1_\mathrm{C}\right\rangle\left\langle 0_\mathrm{C}\right| + \mathrm{H.c.}\right)\right], \quad (1)$$

$$R_{\pm\mathrm{Z}}(\chi,\phi) = \exp\left[-\frac{i\chi}{2}\left(e^{-i\phi}\left|\pm\mathrm{Z}_\mathrm{C}\right\rangle\left\langle 0_\mathrm{C}\right| + \mathrm{H.c.}\right)\right]. \quad (2)$$

Here the carrier operation $R_0(\chi,\phi)$ drives the coupling $|0_\mathrm{C}\rangle \leftrightarrow |1_\mathrm{C}\rangle$, meanwhile the Zeeman operations $R_{\pm\mathrm{Z}}(\chi,\phi)$ drive the coupling $|0_\mathrm{C}\rangle \leftrightarrow |\pm\mathrm{Z}_\mathrm{C}\rangle$ resonantly. Coupling with X and Y are enabled by counter-propagating Raman laser beams, which execute blue side-band operations between hyperfine and motional levels

$$R_\mathrm{X}(\chi,\phi) = \exp\left[\frac{\chi}{2}\left(e^{-i\phi}\sigma_+ a_\mathrm{X}^\dagger - e^{i\phi}\sigma_- a_\mathrm{X}\right)\right], \quad (3)$$

$$R_\mathrm{Y}(\chi,\phi) = \exp\left[\frac{\chi}{2}\left(e^{-i\phi}\sigma_+ a_\mathrm{Y}^\dagger - e^{i\phi}\sigma_- a_\mathrm{Y}\right)\right], \quad (4)$$

where $\sigma_+ = |1_\mathrm{C}\rangle\langle 0_\mathrm{C}|$, $\sigma_- = |0_\mathrm{C}\rangle\langle 1_\mathrm{C}|$, a_X ($a_\mathrm{X}^\dagger$) and a_Y ($a_\mathrm{Y}^\dagger$) are the annihilation (creation) operators of motional modes X and Y. We proceed to describe the experimental details for realizing (i) preprocessing, (ii) postprocessing and (iii) the actions of the server.

Preprocessing – We engineer the required mixed state ρ_1 by building 4 separate 3-qubit quantum circuits $\mathcal{C}_{l,m}$, such that the action of $\mathcal{C}_{l,m}$ converts $|0\rangle_C\,|0\rangle_X\,|0\rangle_Y$ to $|\phi_{l,m}\rangle$, where

$$|\psi_{l,m}\rangle = (|0_\mathrm{C} l_\mathrm{X} m_\mathrm{Y}\rangle + |1_\mathrm{C} m_\mathrm{X} l_\mathrm{Y}\rangle)/\sqrt{2}. \quad (5)$$

A diagram of each circuit is depicted in Supplementary Fig. 1. Algebraically, the actions can be described as

$$|0_\mathrm{C}0_\mathrm{X}0_\mathrm{Y}\rangle \rightarrow \frac{|0_\mathrm{C}\rangle + |1_\mathrm{C}\rangle}{\sqrt{2}}|0_\mathrm{X}\rangle|0_\mathrm{Y}\rangle = |\psi'_{0,0}\rangle, \quad (6)$$

$$|0_\mathrm{C}0_\mathrm{X}0_\mathrm{Y}\rangle \rightarrow \frac{|0_\mathrm{C}\rangle + |1_\mathrm{C}\rangle}{\sqrt{2}}|0_\mathrm{X}\rangle|0_\mathrm{Y}\rangle \quad (7)$$

$$\rightarrow |\psi'_{0,1}\rangle = \frac{|0_\mathrm{C}0_\mathrm{X}1_\mathrm{Y}\rangle - |1_\mathrm{C}1_\mathrm{X}0_\mathrm{Y}\rangle}{\sqrt{2}}, \quad (8)$$

$$|0_\mathrm{C}0_\mathrm{X}0_\mathrm{Y}\rangle \rightarrow \frac{|0_\mathrm{C}\rangle + |1_\mathrm{C}\rangle}{\sqrt{2}}|0_\mathrm{X}\rangle|0_\mathrm{Y}\rangle \quad (9)$$

$$\rightarrow |\psi'_{1,0}\rangle = \frac{|0_\mathrm{C}1_\mathrm{X}0_\mathrm{Y}\rangle + |1_\mathrm{C}0_\mathrm{X}1_\mathrm{Y}\rangle}{\sqrt{2}}, \quad (10)$$

$$|0_\mathrm{C}0_\mathrm{X}0_\mathrm{Y}\rangle \rightarrow |0_\mathrm{C}1_\mathrm{X}1_\mathrm{Y}\rangle \quad (11)$$

$$\rightarrow |\psi'_{1,1}\rangle = \frac{|0_\mathrm{C}\rangle + |1_\mathrm{C}\rangle}{\sqrt{2}}|1_\mathrm{X}\rangle|1_\mathrm{Y}\rangle. \quad (12)$$

An equal statistical mixing of these four states then gives us the mixed state

$$\rho'_1 = \frac{1}{4}\sum_{l,m=0}^{1}|\psi'_{l,m}\rangle\langle\psi'_{l,m}| = Z_1^\dagger \rho_1 Z_1, \quad (13)$$

where the phase shift

$$Z_1 = \mathrm{Diag}\,(1,1,1,1,1,-1,1,1), \quad (14)$$

caused by the minus sign of $|\psi'_{0,1}\rangle$ in Supplementary Eq. (8), has no effect on the results (See Supplementary Note 2). The detailed of implementation of each process is shown in Supplementary Tab. 1.

The final element of the preprocessing is to map information between X and C, for delivery to the server. In

Supplementary Table 1. **Implementation of preprocessing.** Each sequence shown in the right column implements the corresponding operation shown in the left column.

Operation	Sequence
$\|0_C 0_X 0_Y\rangle \to (\|0_C 0_X 0_Y\rangle + \|1_C 0_X 0_Y\rangle)/\sqrt{2}$	$R_0(\pi/2, -\pi/2)$
$\|0_C 0_X 0_Y\rangle \to (\|0_C 0_X 1_Y\rangle - \|1_C 1_X 0_Y\rangle)/\sqrt{2}$	$R_0(\pi/2, -\pi/2)$, $R_0(\pi, \pi/2)$, $R_{-Z}(\pi, 0)$, $R_0(\pi, -\pi/2)$, $R_Y(\pi, 0)$, $R_0(\pi, \pi/2)$, $R_{-Z}(\pi, 0)$, $R_X(\pi, 0)$, $R_{-Z}(\pi, \pi)$
$\|0_C 0_X 0_Y\rangle \to (\|0_C 1_X 0_Y\rangle + \|1_C 0_X 1_Y\rangle)/\sqrt{2}$	$R_0(\pi/2, -\pi/2)$, $R_0(\pi, \pi/2)$, $R_{-Z}(\pi, 0)$, $R_0(\pi, -\pi/2)$, $R_X(\pi, 0)$, $R_0(\pi, \pi/2)$, $R_{-Z}(\pi, 0)$, $R_Y(\pi, \pi)$, $R_{-Z}(\pi, \pi)$
$\|0_C 0_X 0_Y\rangle \to (\|0_C 1_X 1_Y\rangle + \|1_C 1_X 1_Y\rangle)/\sqrt{2}$	$R_X(\pi, 0)$, $R_0(\pi, \pi/2)$, $R_Y(\pi, \pi)$, $R_0(\pi, \pi/2)$, $R_0(\pi/2, -\pi/2)$

Supplementary Table 2. **Implementation of postprocessing.** Here $\alpha = \arccos\left[\csc(\pi/\sqrt{2})/\sqrt{2}\right]$ and $\gamma = \phi - \arccos\left[\cot(\pi/\sqrt{2})\right]$.

Operation	Sequence
$F(\phi)$	$R_{-Z}(\pi, 0)$, $R_Y(\pi, \pi)$, $R_X(\pi/\sqrt{2}, \gamma)$, $R_X(\pi/\sqrt{2}, 2\alpha + \gamma)$, $R_Z(\pi, \pi)$, $R_{-Z}(\pi, \pi)$, $R_Z(\pi, 0)$, $R_X(\pi/\sqrt{2}, 2\alpha + \gamma)$, $R_X(\pi/\sqrt{2}, \gamma)$, $R_Y(\pi, 0)$, $R_Z(\pi, \pi)$

Supplementary Table 3. **Implementation of modified SWAP gate.** Here $\alpha = \arccos\left[\csc(\pi/\sqrt{2})\sin(\chi/4)\right]$ and $\gamma = \phi - \arccos\left[\cot(\pi/\sqrt{2})\tan(\chi/4)\right]$.

Operation	Sequence
$U(\chi, \theta, \phi)$	$R_0(\pi/2 - \theta, \phi + \pi/2)$, $R_0(\chi, \phi)$, $R_0(\pi/2 - \theta, \phi - \pi/2)$
$S_X(\chi, \phi)$	$R_X(\pi/\sqrt{2}, \gamma)$, $R_X(\sqrt{2}\pi, 2\alpha + \gamma)$, $R_X(\pi/\sqrt{2}, \gamma)$

the theoretical protocol, this is achieved by a SWAP gate between X and C. While this gate is difficult to achieve in our ion trap setup, we developed a suitable sequence of Raman operations (see Supplementary Tab. 3) that implement the class of two-qubits operations

$$S_X(\chi, \phi) = \begin{pmatrix} \cos\frac{\chi}{2} & & & -\sin\frac{\chi}{2}e^{i\phi} \\ & 1 & & \\ & & 1 & \\ \sin\frac{\chi}{2}e^{-i\phi} & & & \cos\frac{\chi}{2} \end{pmatrix} \quad (15)$$

with basis $|0_C 0_X\rangle$, $|1_C 0_X\rangle$, $|0_C 1_X\rangle$ and $|1_C 1_X\rangle$ that works as a suitable replacement – provided a suitably modified SWAP gate is also used during post-processing.

To see this, let U_C denotes action of U on qubit C, U_X the action of U on qubit X. We then observe that

$$S_X(\pi, \pi) U_C S_X(\pi, 0) = Z_2 U_X^* Z_2^\dagger, \quad (16)$$

where the phase shift

$$Z_2 = \mathrm{Diag}\,(1, -1, 1, 1) \quad (17)$$

has no effect on measurement results (See Supplementary Note 2 for proof), and U^* has the same effect on the results as U, since $|\mathrm{tr}(U^*)| = |\mathrm{tr}(U)|$. Thus during preprocessing stage, Alice replaces the SWAP gate with the gate $S_X(\pi, 0)$. Once done, Alice can out-source qubit C to a third party for realization of U.

Postprocessing – The postprocessing module consists of two steps. The first is to perform a suitably modified SWAP gate between C and X. This is done by realization of the gate $S_X(\pi, \pi)$ (again using the pulse sequence given in Supplementary Tab. 3).

The second step is application of a CSWAP gate on X and Y using qubit C as a control. To do this, we first develop a means of implementing the following 3 qubit gate

$$F(\phi) = \begin{pmatrix} 1 & & & & & & & \\ & 1 & & & & & & \\ & & 1 & & & & & \\ & & & 1 & & & & \\ & & & & 1 & & & \\ & & & & & & -e^{i\phi} & \\ & & & & & e^{-i\phi} & & \\ & & & & & & & 1 \end{pmatrix} \quad (18)$$

with basis $|0_C 0_X 0_Y\rangle$, $|0_C 0_X 1_Y\rangle$, $\cdots$, $|1_C 1_X 1_Y\rangle$. Specifically, when qubit C is in state $|0_C\rangle$, it is temporarily shelved into Zeeman levels $|\pm Z_C\rangle$. We first transfer $|0_C\rangle$ to $|-Z_C\rangle$ by $R_{-Z}(\pi, 0)$ in the beginning. At halfway, $|-Z_C\rangle$ is transferred to $|Z_C\rangle$ by sequence $R_Z(\pi, \pi)$, $R_{-Z}(\pi, \pi)$, $R_Z(\pi, 0)$. Finally, we transfer $|Z_C\rangle$ back to $|0_C\rangle$ by $R_Z(\pi, \pi)$ in the end. When qubit C is in state $|1_C\rangle$, a swap of populations between $|1_C 0_X 1_Y\rangle$ and $|1_C 1_X 0_Y\rangle$ is executed by sequence $R_Y(\pi, \pi)$, $S_X(\pi, \phi)$, $R_Y(\pi, 0)$. The full implementation of $F(\phi)$ is shown in Supplementary Tab. 2.

During postprocessing, we perform operation $F(0) = F_0 Z_3$, where F_0 is a standard CSWAP gate, and the phase shift

$$Z_3 = \mathrm{Diag}(1, 1, 1, 1, 1, -1, 1, 1) \quad (19)$$

has no effect on the results (proved in Supplementary Note 2).

The final step – a σ_1 measurement on qubit C – is realized by sequentially applying Hadamard gate and standard fluorescence detection.

Benchmarking – To benchmark the modular DQC1 protocol, we take on the role of the server, Bob, who is out-sourced by Alice to apply a unitary U on qubit C before returning it to Alice for postprocessing.

To test modularity, we needed to ensure that Alice's device could correctly estimate $|T(U)|$ for any possible U without modification. As such, in experiment, our simulation of the server needs to synthesize a wide variety of possible U. To do this, we developed a generic scheme for synthesizing

$$U = \exp\left(-i\frac{\chi}{2}\sigma_{\theta,\phi}\right) \tag{20}$$

where

$$\sigma_{\theta,\phi} = \begin{pmatrix} -\cos\theta & \sin\theta e^{i\phi} \\ \sin\theta e^{-i\phi} & \cos\theta \end{pmatrix} \tag{21}$$

for general values of χ, θ and ϕ using microwave operations (see Supplementary Tab. 3).

In the experiment, we benchmarked Alice for 19 different choices of U. For each choice, we executed 1000 runs for each possible choice of l and m, resulting in a total of 4000 runs. Thus in total, the experiment involved $4 \times 19 \times 1000 = 76000$ measurements.

Potential Scalability – Here we outline a potential means for scaling our postprocessing and preprocessing modules so that they may be used to build a modular DQC1 algorithm that evaluates $|T(U)|$ for some $2^n \times 2^n$ unitary. Recall that such a algorithm involves 1 control qubit C, together with two 2^n-dimensional registers, X and Y.

Consider encoding each 2^n-dimensional register to the ground and first excited states of n motional modes. For each 2^n-dimensional register, we can label the elements of a complete basis by n-bit binary strings, such that the X register basis is indexed by $l = l_{n-1}\cdots l_1 l_0$ and the Y register is indexed by $m = m_{n-1}\cdots m_1 m_0$.

To scale the preprocessing module up, we start by generating two n-bit binary strings at random, then we synthesize the circuit $\mathcal{C}_{l,m}$ described by Supplementary Fig. 2. Meanwhile the postprocessing module can be scaled up to the general case of two 2^n-dimensional registers by repeatedly conducting $F(0)$ (see Supplementary Fig. 3).

If Alice repeats the above for each call to the server, and after K calls (where K scales as polynomial of n), we are then able to estimate $|T(U)|$ to some fixed accuracy.

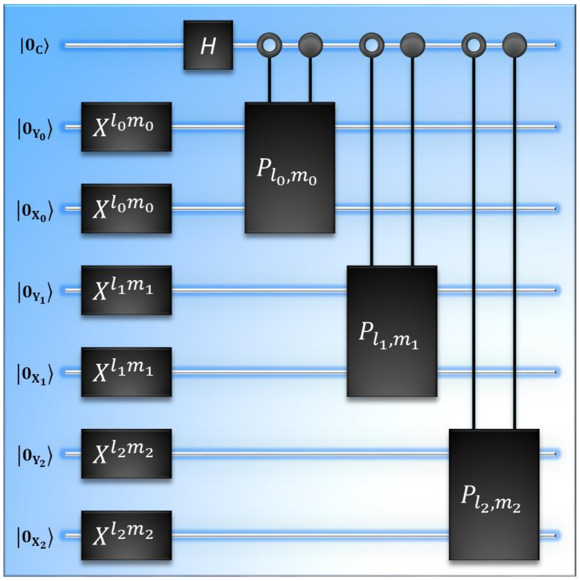

Supplementary Figure 2. **The circuit $\mathcal{C}_{l,m}$ realizing a scalable version of the preprocessing module.** In this picture the n-qubits in registers X and Y have been interlaced so that the order of representation is qubit 0 of register Y, qubit 0 of register X, qubit 1 of register Y, qubit 1 of register X, etc. The first step of the general preprocessing module involves generating two n-bit strings $l = l_{n-1}\cdots l_1 l_0$ and $m = m_{n-1}\cdots m_1 m_0$ at random. Afterwards we take the i-th qubit of each register and apply the not gate to the power of $l_i \times m_i$ to both qubits. This gate $X^{l_i m_i}$ flips the target qubit whenever $l_i = m_i = 1$. We also apply the Hadamard gate H, locally to the control qubit. Finally we implement the gate P_{l_i,m_i} between the i-th qubit of each register and the control, where $P_{0,0}$, $P_{0,1}$, $P_{1,0}$ and $P_{1,1}$ are defined in Supplementary Fig. 1.

SUPPLEMENTARY NOTE 2: INVARIANCE TO PHASE SHIFTS

Observe that in our implementation, many operations are synthesized up to some phase shift. Here we prove that these phase shifts, namely the phase gates, Z_1, Z_2 and Z_3 (see Supplementary Eqs. (14)(17)(19)), have no effect on theoretical expectation value $\langle\sigma_1\rangle$. In fact, the above conclusion is true for general n, as long as $Z_1 = Z_3$.

In this case, the state before measurement is

$$\begin{aligned}
\rho_3' = {} & F_0 Z_1 Z_2 U_X Z_2^\dagger Z_1^\dagger F_0 \begin{pmatrix} I & I \\ I & I \end{pmatrix} \\
& F_0 Z_1 Z_2 U_X^\dagger Z_2^\dagger Z_1^\dagger F_0 / N' \\
= {} & \tfrac{1}{N'} \Lambda F_0 U_X F_0 \Lambda^\dagger \begin{pmatrix} I & I \\ I & I \end{pmatrix} \Lambda F_0 U_X^\dagger F_0 \Lambda^\dagger \\
= {} & \begin{pmatrix} \Lambda_0 U_X \Lambda_0^\dagger & \\ & \Lambda_1 U_Y \Lambda_1^\dagger \end{pmatrix} \begin{pmatrix} I & I \\ I & I \end{pmatrix} \\
& \begin{pmatrix} \Lambda_0 U_X^\dagger \Lambda_0^\dagger & \\ & \Lambda_1 U_Y^\dagger \Lambda_1^\dagger \end{pmatrix} / N' \\
= {} & \tfrac{1}{N'} \begin{pmatrix} I & \Lambda_0 U_X \Lambda_0^\dagger \Lambda_1 U_Y^\dagger \Lambda_1^\dagger \\ \Lambda_1 U_Y \Lambda_1^\dagger \Lambda_0 U_X^\dagger \Lambda_0^\dagger & I \end{pmatrix}
\end{aligned} \tag{22}$$

with basis $|0_C\rangle$ and $|1_C\rangle$, where I is the identity matrix,

$$\Lambda = F_0 Z_1 Z_2 F_0 = \begin{pmatrix} \Lambda_0 & \\ & \Lambda_1 \end{pmatrix} \tag{23}$$

has only eigenvalues of ± 1, and N' is the dimension of system. For general n, the client possesses 1 control qubit

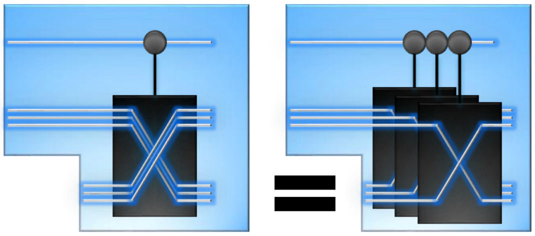

Supplementary Figure 3. **Scalability of the CSWAP gate in the postprocessing module.** A CSWAP gate with n-qubit registers, as shown in left circuit, is equivalent to a combination of n CSWAP gates with 1-qubit registers, as shown in right circuit.

and 2 registers, and the server possesses 1 register. Thus $N' = 2N^3$. In our special case, the control qubit serves as server register. Thus $N' = 8$.

We derive the expectation value $\langle\sigma_1\rangle$ of ρ_3'

$$\begin{aligned}\langle\sigma_1\rangle =&\ \mathrm{tr}\left(\Lambda_0 U_{\mathrm{X}} \Lambda_0^\dagger \Lambda_1 U_{\mathrm{Y}}^\dagger \Lambda_1^\dagger + \mathrm{h.c.}\right)/N' \\ =&\ \textstyle\sum_{l,m} \langle l|\, \Lambda_0 U_{\mathrm{X}} \Lambda_0^\dagger \,|m\rangle \langle m|\, \Lambda_1 U_{\mathrm{Y}}^\dagger \Lambda_1^\dagger \,|l\rangle /N' + \mathrm{h.c.} \\ =&\ \textstyle\sum_m \langle m|\, U_{\mathrm{X}} \,|m\rangle \langle m|\, U_{\mathrm{Y}}^\dagger \,|m\rangle /N' + \mathrm{h.c.} \\ =&\ \mathrm{tr}(U \otimes U^\dagger + U^\dagger \otimes U)/(2N) \\ =&\ |\mathrm{tr}(U)/N|^2,\end{aligned} \tag{24}$$

where $|l\rangle = |m\rangle$ (if $|l\rangle \neq |m\rangle$, then $\langle l|\, U_{\mathrm{X}} \,|m\rangle = 0$ or $\langle l|\, U_{\mathrm{Y}} \,|m\rangle = 0$) traverses the N' eigenstates of Λ, and U is an $N \times N$ matrix. Thus we prove our conclusion.

SUPPLEMENTARY NOTE 3: PERFORMANCE OF CSWAP GATE

The theoretical result of $F(0)$ is defined in Supplementary Eq. (18). In experiment, we obtain the absolute value of each matrix element of $F(0)$. We also obtain the phase of any element that has a absolute value of 1 in theory. These are all done by measuring population $|\langle\varphi|\, F(0) \,|\psi\rangle|^2$ for necessary inputs $|\psi\rangle$ and outputs $|\varphi\rangle$. The resulting outcomes are depicted in Supplementary Fig. 4. Each measurement

$$|\langle\varphi|\, F(0) \,|\psi\rangle|^2 = |\langle 1_{\mathrm{C}}1_{\mathrm{X}}1_{\mathrm{Y}}|\, R_{\mathrm{o}} F(0) R_{\mathrm{i}} \,|0_{\mathrm{C}}0_{\mathrm{X}}0_{\mathrm{Y}}\rangle|^2 \tag{25}$$

consists of the foolowing 5 steps. First, we prepare $|0_{\mathrm{C}}0_{\mathrm{X}}0_{\mathrm{Y}}\rangle$ by standard sideband cooling. Second, we conduct R_{i}, which prepares $|\psi\rangle$ from $|0_{\mathrm{C}}0_{\mathrm{X}}0_{\mathrm{Y}}\rangle$ (see Supplementary Tab. 4). Third is $F(0)$. Fourth is R_{i}, which transforms output $|\varphi\rangle$ to $|1_{\mathrm{C}}1_{\mathrm{X}}1_{\mathrm{Y}}\rangle$ (see Supplementary Tab. 5). And the last is the population measurement of $|1_{\mathrm{C}}1_{\mathrm{X}}1_{\mathrm{Y}}\rangle$ (see Supplementary Tab. 6).

To measure the population of $|1_{\mathrm{C}}1_{\mathrm{X}}1_{\mathrm{Y}}\rangle$, we develop $P_{\mathrm{X}}(\phi)$ operation that instigates π transitions for both $|0_{\mathrm{C}}0_{\mathrm{X}}\rangle \leftrightarrow |1_{\mathrm{C}}1_{\mathrm{X}}\rangle$ and $|0_{\mathrm{C}}1_{\mathrm{X}}\rangle \leftrightarrow |1_{\mathrm{C}}2_{\mathrm{X}}\rangle$, and $P_{\mathrm{Y}}(\phi)$ operation which is defined similarly (see Supplementary Tab. 6). By a proper sequence that involves $P_{\mathrm{X}}(0)$ and

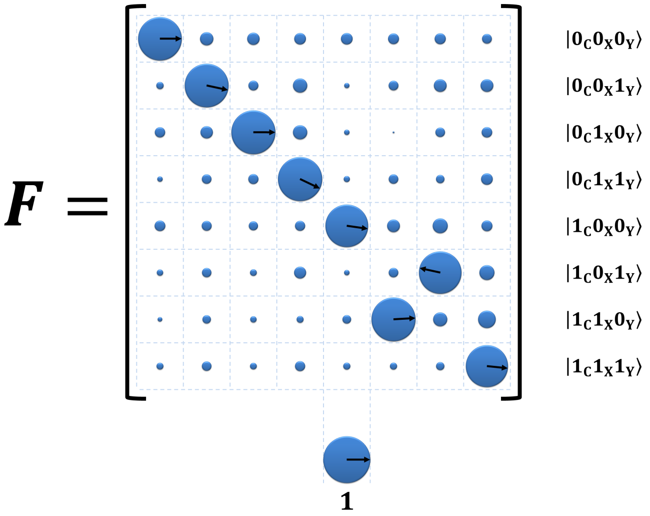

Supplementary Figure 4. **The truth table of control SWAP gate.** Visual representation of relevant probabilities and the phases of the CSWAP gate in the computational basis. The area of the orange disk on the lth column and m^{th} row reflects the probability of obtaining corresponding output $|l\rangle$ given corresponding input $|m\rangle$, where l, m range over all binary representations of the 3 encoded qubits. So the radius of the disk is proportional to $|\langle l|\, F \,|m\rangle|$. Meanwhile, the orientation of each black arrow on the disk gives the phase information of corresponding element $\langle l|\, F \,|m\rangle$. The radius and orientation of the blue disk represents an amplitude of 1 and a phase of 0. Note that the negative phase $\langle 1_{\mathrm{C}}0_{\mathrm{X}}1_{\mathrm{Y}}|\, F \,|1_{\mathrm{C}}1_{\mathrm{X}}0_{\mathrm{Y}}\rangle = -1$ is consequence of the choice of physical realization, and does not affect computational output (see Supplementary Note 2).

$P_{\mathrm{Y}}(0)$, we are able to transforms $|1_{\mathrm{C}}1_{\mathrm{X}}1_{\mathrm{Y}}\rangle$ to $|0_{\mathrm{C}}\rangle$, and $|0_{\mathrm{C}}0_{\mathrm{X}}0_{\mathrm{Y}}\rangle$, $|0_{\mathrm{C}}0_{\mathrm{X}}1_{\mathrm{Y}}\rangle$, $\cdots$, $|1_{\mathrm{C}}1_{\mathrm{X}}0_{\mathrm{Y}}\rangle$ to $|1_{\mathrm{C}}\rangle$ (see Supplementary Tab. 6). After this sequence, $|1_{\mathrm{C}}1_{\mathrm{X}}1_{\mathrm{Y}}\rangle$ population measurement can then be completed by measuring the population of $|0_{\mathrm{C}}\rangle$, which is realized by standard fluorescence detection and detection error correction. We note that, our method of $|1_{\mathrm{C}}1_{\mathrm{X}}1_{\mathrm{Y}}\rangle$ measurement works only for the state that not populates $|(l > 1)_{\mathrm{X}}\rangle$ and $|(m > 1)_{\mathrm{Y}}\rangle$. Our protocol naturally ensures that $F(0)\,|\psi\rangle$ fulfills this condition. To make $R_{\mathrm{o}}F(0)\,|\psi\rangle$ still fulfills, we cannot use R_{X} and R_{Y} for the implementation of R_{o}. Instead, we use S_{X} (see Supplementary Eq. (15)), and S_{Y}, which works on Y mode and qubit C similar to S_{X}.

In experiment, we first let $|\varphi\rangle$ and $|\psi\rangle$ traverse $|0_{\mathrm{C}}0_{\mathrm{X}}0_{\mathrm{Y}}\rangle$, $|0_{\mathrm{C}}0_{\mathrm{X}}1_{\mathrm{Y}}\rangle$, $\cdots$, $|1_{\mathrm{C}}1_{\mathrm{X}}1_{\mathrm{Y}}\rangle$. Hence, we obtain the absolute value of each matrix element of $F(0)$ by square root. Then we obtain the phase of any element as follows. For any base states $|l\rangle$ and $|k\rangle$, by letting $|\varphi\rangle$ traverse $|l\rangle$, $|k\rangle$, $(|l\rangle + |k\rangle)/\sqrt{2}$ and $(|l\rangle + i\,|k\rangle)/\sqrt{2}$, we

Supplementary Table 4. **Implementation of R_i.**

Operation	Sequence
$\|0_\mathrm{C}0_\mathrm{X}0_\mathrm{Y}\rangle \to \|0_\mathrm{C}0_\mathrm{X}1_\mathrm{Y}\rangle$	$R_\mathrm{Y}(\pi,0)$, $R_0(\pi,\pi/2)$
$\|0_\mathrm{C}0_\mathrm{X}0_\mathrm{Y}\rangle \to \|0_\mathrm{C}1_\mathrm{X}0_\mathrm{Y}\rangle$	$R_\mathrm{X}(\pi,0)$, $R_0(\pi,\pi/2)$
$\|0_\mathrm{C}0_\mathrm{X}0_\mathrm{Y}\rangle \to \|0_\mathrm{C}1_\mathrm{X}1_\mathrm{Y}\rangle$	$R_\mathrm{X}(\pi,0)$, $R_0(\pi,\pi/2)$, $R_\mathrm{Y}(\pi,0)$, $R_0(\pi,\pi/2)$
$\|0_\mathrm{C}0_\mathrm{X}0_\mathrm{Y}\rangle \to \|1_\mathrm{C}0_\mathrm{X}0_\mathrm{Y}\rangle$	$R_0(\pi,-\pi/2)$
$\|0_\mathrm{C}0_\mathrm{X}0_\mathrm{Y}\rangle \to \|1_\mathrm{C}0_\mathrm{X}1_\mathrm{Y}\rangle$	$R_\mathrm{Y}(\pi,0)$
$\|0_\mathrm{C}0_\mathrm{X}0_\mathrm{Y}\rangle \to \|1_\mathrm{C}1_\mathrm{X}0_\mathrm{Y}\rangle$	$R_\mathrm{X}(\pi,0)$
$\|0_\mathrm{C}0_\mathrm{X}0_\mathrm{Y}\rangle \to \|1_\mathrm{C}1_\mathrm{X}1_\mathrm{Y}\rangle$	$R_\mathrm{X}(\pi,0)$, $R_0(\pi,\pi/2)$, $R_\mathrm{Y}(\pi,0)$
$\|0_\mathrm{C}0_\mathrm{X}0_\mathrm{Y}\rangle \to (\|0_\mathrm{C}0_\mathrm{X}0_\mathrm{Y}\rangle + \|1_\mathrm{C}0_\mathrm{X}0_\mathrm{Y}\rangle)/\sqrt{2}$	$R_0(\pi/2,-\pi/2)$
$\|0_\mathrm{C}0_\mathrm{X}0_\mathrm{Y}\rangle \to (\|0_\mathrm{C}0_\mathrm{X}0_\mathrm{Y}\rangle + \|1_\mathrm{C}0_\mathrm{X}1_\mathrm{Y}\rangle)/\sqrt{2}$	$R_\mathrm{Y}(\pi/2,0)$
$\|0_\mathrm{C}0_\mathrm{X}0_\mathrm{Y}\rangle \to (\|0_\mathrm{C}0_\mathrm{X}0_\mathrm{Y}\rangle + \|1_\mathrm{C}1_\mathrm{X}0_\mathrm{Y}\rangle)/\sqrt{2}$	$R_\mathrm{X}(\pi/2,0)$
$\|0_\mathrm{C}0_\mathrm{X}0_\mathrm{Y}\rangle \to (\|0_\mathrm{C}0_\mathrm{X}1_\mathrm{Y}\rangle + \|1_\mathrm{C}1_\mathrm{X}0_\mathrm{Y}\rangle)/\sqrt{2}$	$R_{-\mathrm{Z}}(\pi/2,-\pi/2)$, $R_\mathrm{Y}(\pi,0)$, $R_0(\pi,\pi/2)$, $R_{-\mathrm{Z}}(\pi,\pi/2)$, $R_\mathrm{X}(\pi,0)$, $R_{-\mathrm{Z}}(\pi,-\pi/2)$
$\|0_\mathrm{C}0_\mathrm{X}0_\mathrm{Y}\rangle \to (\|0_\mathrm{C}0_\mathrm{X}1_\mathrm{Y}\rangle + \|1_\mathrm{C}1_\mathrm{X}1_\mathrm{Y}\rangle)/\sqrt{2}$	$R_\mathrm{Y}(\pi,0)$, $R_0(\pi,\pi/2)$, $R_\mathrm{X}(\pi/2,0)$
$\|0_\mathrm{C}0_\mathrm{X}0_\mathrm{Y}\rangle \to (\|0_\mathrm{C}1_\mathrm{X}0_\mathrm{Y}\rangle + \|1_\mathrm{C}0_\mathrm{X}1_\mathrm{Y}\rangle)/\sqrt{2}$	$R_{-\mathrm{Z}}(\pi/2,-\pi/2)$, $R_\mathrm{X}(\pi,0)$, $R_0(\pi,\pi/2)$, $R_{-\mathrm{Z}}(\pi,\pi/2)$, $R_\mathrm{Y}(\pi,0)$, $R_{-\mathrm{Z}}(\pi,-\pi/2)$
$\|0_\mathrm{C}0_\mathrm{X}0_\mathrm{Y}\rangle \to (\|0_\mathrm{C}1_\mathrm{X}0_\mathrm{Y}\rangle + \|1_\mathrm{C}1_\mathrm{X}1_\mathrm{Y}\rangle)/\sqrt{2}$	$R_\mathrm{X}(\pi,0)$, $R_0(\pi,\pi/2)$, $R_\mathrm{Y}(\pi/2,0)$
$\|0_\mathrm{C}0_\mathrm{X}0_\mathrm{Y}\rangle \to (\|0_\mathrm{C}1_\mathrm{X}1_\mathrm{Y}\rangle + \|1_\mathrm{C}1_\mathrm{X}1_\mathrm{Y}\rangle)/\sqrt{2}$	$R_\mathrm{X}(\pi,0)$, $R_0(\pi,\pi/2)$, $R_\mathrm{Y}(\pi,0)$, $R_0(\pi/2,\pi/2)$
$\|0_\mathrm{C}0_\mathrm{X}0_\mathrm{Y}\rangle \to (\|0_\mathrm{C}0_\mathrm{X}0_\mathrm{Y}\rangle + i\,\|1_\mathrm{C}0_\mathrm{X}0_\mathrm{Y}\rangle)/\sqrt{2}$	$R_0(\pi/2,\pi)$
$\|0_\mathrm{C}0_\mathrm{X}0_\mathrm{Y}\rangle \to (\|0_\mathrm{C}0_\mathrm{X}0_\mathrm{Y}\rangle + i\,\|1_\mathrm{C}0_\mathrm{X}1_\mathrm{Y}\rangle)/\sqrt{2}$	$R_\mathrm{Y}(\pi/2,-\pi/2)$
$\|0_\mathrm{C}0_\mathrm{X}0_\mathrm{Y}\rangle \to (\|0_\mathrm{C}0_\mathrm{X}0_\mathrm{Y}\rangle + i\,\|1_\mathrm{C}1_\mathrm{X}0_\mathrm{Y}\rangle)/\sqrt{2}$	$R_\mathrm{X}(\pi/2,-\pi/2)$
$\|0_\mathrm{C}0_\mathrm{X}0_\mathrm{Y}\rangle \to (\|0_\mathrm{C}0_\mathrm{X}1_\mathrm{Y}\rangle + i\,\|1_\mathrm{C}1_\mathrm{X}0_\mathrm{Y}\rangle)/\sqrt{2}$	$R_{-\mathrm{Z}}(\pi/2,-\pi/2)$, $R_\mathrm{Y}(\pi,0)$, $R_0(\pi,\pi/2)$, $R_{-\mathrm{Z}}(\pi,\pi/2)$, $R_\mathrm{X}(\pi,-\pi/2)$, $R_{-\mathrm{Z}}(\pi,-\pi/2)$
$\|0_\mathrm{C}0_\mathrm{X}0_\mathrm{Y}\rangle \to (\|0_\mathrm{C}0_\mathrm{X}1_\mathrm{Y}\rangle + i\,\|1_\mathrm{C}1_\mathrm{X}1_\mathrm{Y}\rangle)/\sqrt{2}$	$R_\mathrm{Y}(\pi,0)$, $R_0(\pi,\pi/2)$, $R_\mathrm{X}(\pi/2,-\pi/2)$
$\|0_\mathrm{C}0_\mathrm{X}0_\mathrm{Y}\rangle \to (\|0_\mathrm{C}1_\mathrm{X}0_\mathrm{Y}\rangle + i\,\|1_\mathrm{C}0_\mathrm{X}1_\mathrm{Y}\rangle)/\sqrt{2}$	$R_{-\mathrm{Z}}(\pi/2,-\pi/2)$, $R_\mathrm{X}(\pi,0)$, $R_0(\pi,\pi/2)$, $R_{-\mathrm{Z}}(\pi,\pi/2)$, $R_\mathrm{Y}(\pi,-\pi/2)$, $R_{-\mathrm{Z}}(\pi,-\pi/2)$
$\|0_\mathrm{C}0_\mathrm{X}0_\mathrm{Y}\rangle \to (\|0_\mathrm{C}1_\mathrm{X}0_\mathrm{Y}\rangle + i\,\|1_\mathrm{C}1_\mathrm{X}1_\mathrm{Y}\rangle)/\sqrt{2}$	$R_\mathrm{X}(\pi,0)$, $R_0(\pi,\pi/2)$, $R_\mathrm{Y}(\pi/2,-\pi/2)$
$\|0_\mathrm{C}0_\mathrm{X}0_\mathrm{Y}\rangle \to (\|0_\mathrm{C}1_\mathrm{X}1_\mathrm{Y}\rangle + i\,\|1_\mathrm{C}1_\mathrm{X}1_\mathrm{Y}\rangle)/\sqrt{2}$	$R_\mathrm{X}(\pi,0)$, $R_0(\pi,\pi/2)$, $R_\mathrm{Y}(\pi,-\pi/2)$, $R_0(\pi/2,0)$

Supplementary Table 5. **Implementation of R_o.**

Operation	Sequence
$\|0_\mathrm{C}0_\mathrm{X}0_\mathrm{Y}\rangle \to \|1_\mathrm{C}1_\mathrm{X}1_\mathrm{Y}\rangle$	$S_\mathrm{X}(\pi,0)$, $R_0(\pi,\pi/2)$, $S_\mathrm{Y}(\pi,0)$
$\|0_\mathrm{C}0_\mathrm{X}1_\mathrm{Y}\rangle \to \|1_\mathrm{C}1_\mathrm{X}1_\mathrm{Y}\rangle$	$S_\mathrm{X}(\pi,0)$
$\|0_\mathrm{C}1_\mathrm{X}0_\mathrm{Y}\rangle \to \|1_\mathrm{C}1_\mathrm{X}1_\mathrm{Y}\rangle$	$S_\mathrm{Y}(\pi,0)$
$\|0_\mathrm{C}1_\mathrm{X}1_\mathrm{Y}\rangle \to \|1_\mathrm{C}1_\mathrm{X}1_\mathrm{Y}\rangle$	$R_0(\pi,-\pi/2)$
$\|1_\mathrm{C}0_\mathrm{X}0_\mathrm{Y}\rangle \to \|1_\mathrm{C}1_\mathrm{X}1_\mathrm{Y}\rangle$	$R_0(\pi,\pi/2)$, $S_\mathrm{X}(\pi,0)$, $R_0(\pi,\pi/2)$, $S_\mathrm{Y}(\pi,0)$
$\|1_\mathrm{C}0_\mathrm{X}1_\mathrm{Y}\rangle \to \|1_\mathrm{C}1_\mathrm{X}1_\mathrm{Y}\rangle$	$R_0(\pi,\pi/2)$, $S_\mathrm{X}(\pi,0)$
$\|1_\mathrm{C}1_\mathrm{X}0_\mathrm{Y}\rangle \to \|1_\mathrm{C}1_\mathrm{X}1_\mathrm{Y}\rangle$	$R_0(\pi,\pi/2)$, $S_\mathrm{Y}(\pi,0)$
$(\|0_\mathrm{C}0_\mathrm{X}0_\mathrm{Y}\rangle + \|1_\mathrm{C}0_\mathrm{X}0_\mathrm{Y}\rangle)/\sqrt{2} \to \|1_\mathrm{C}1_\mathrm{X}1_\mathrm{Y}\rangle$	$R_0(\pi/2,\pi/2)$, $S_\mathrm{X}(\pi,0)$, $R_0(\pi,\pi/2)$, $S_\mathrm{Y}(\pi,0)$
$(\|0_\mathrm{C}0_\mathrm{X}0_\mathrm{Y}\rangle + \|1_\mathrm{C}0_\mathrm{X}1_\mathrm{Y}\rangle)/\sqrt{2} \to \|1_\mathrm{C}1_\mathrm{X}1_\mathrm{Y}\rangle$	$S_\mathrm{Y}(\pi/2,0)$, $R_0(\pi,\pi/2)$, $S_\mathrm{X}(\pi,0)$
$(\|0_\mathrm{C}0_\mathrm{X}0_\mathrm{Y}\rangle + \|1_\mathrm{C}1_\mathrm{X}0_\mathrm{Y}\rangle)/\sqrt{2} \to \|1_\mathrm{C}1_\mathrm{X}1_\mathrm{Y}\rangle$	$S_\mathrm{X}(\pi/2,0)$, $R_0(\pi,\pi/2)$, $S_\mathrm{Y}(\pi,0)$
$(\|0_\mathrm{C}0_\mathrm{X}1_\mathrm{Y}\rangle + \|1_\mathrm{C}0_\mathrm{X}1_\mathrm{Y}\rangle)/\sqrt{2} \to \|1_\mathrm{C}1_\mathrm{X}1_\mathrm{Y}\rangle$	$R_0(\pi/2,\pi/2)$, $S_\mathrm{X}(\pi,0)$
$(\|0_\mathrm{C}0_\mathrm{X}1_\mathrm{Y}\rangle + \|1_\mathrm{C}1_\mathrm{X}1_\mathrm{Y}\rangle)/\sqrt{2} \to \|1_\mathrm{C}1_\mathrm{X}1_\mathrm{Y}\rangle$	$S_\mathrm{X}(\pi/2,0)$
$(\|0_\mathrm{C}1_\mathrm{X}0_\mathrm{Y}\rangle + \|1_\mathrm{C}1_\mathrm{X}0_\mathrm{Y}\rangle)/\sqrt{2} \to \|1_\mathrm{C}1_\mathrm{X}1_\mathrm{Y}\rangle$	$R_0(\pi/2,\pi/2)$, $S_\mathrm{Y}(\pi,0)$
$(\|0_\mathrm{C}1_\mathrm{X}0_\mathrm{Y}\rangle + \|1_\mathrm{C}1_\mathrm{X}1_\mathrm{Y}\rangle)/\sqrt{2} \to \|1_\mathrm{C}1_\mathrm{X}1_\mathrm{Y}\rangle$	$S_\mathrm{Y}(\pi/2,0)$
$(\|0_\mathrm{C}1_\mathrm{X}1_\mathrm{Y}\rangle + \|1_\mathrm{C}1_\mathrm{X}1_\mathrm{Y}\rangle)/\sqrt{2} \to \|1_\mathrm{C}1_\mathrm{X}1_\mathrm{Y}\rangle$	$R_0(\pi/2,-\pi/2)$
$(\|0_\mathrm{C}0_\mathrm{X}0_\mathrm{Y}\rangle + i\,\|1_\mathrm{C}0_\mathrm{X}0_\mathrm{Y}\rangle)/\sqrt{2} \to \|1_\mathrm{C}1_\mathrm{X}1_\mathrm{Y}\rangle$	$R_0(\pi/2,0)$, $S_\mathrm{X}(\pi,0)$, $R_0(\pi,\pi/2)$, $S_\mathrm{Y}(\pi,0)$
$(\|0_\mathrm{C}0_\mathrm{X}0_\mathrm{Y}\rangle + i\,\|1_\mathrm{C}0_\mathrm{X}1_\mathrm{Y}\rangle)/\sqrt{2} \to \|1_\mathrm{C}1_\mathrm{X}1_\mathrm{Y}\rangle$	$S_\mathrm{Y}(\pi/2,-\pi/2)$, $R_0(\pi,\pi/2)$, $S_\mathrm{X}(\pi,0)$
$(\|0_\mathrm{C}0_\mathrm{X}0_\mathrm{Y}\rangle + i\,\|1_\mathrm{C}1_\mathrm{X}0_\mathrm{Y}\rangle)/\sqrt{2} \to \|1_\mathrm{C}1_\mathrm{X}1_\mathrm{Y}\rangle$	$S_\mathrm{X}(\pi/2,-\pi/2)$, $R_0(\pi,\pi/2)$, $S_\mathrm{Y}(\pi,0)$
$(\|0_\mathrm{C}0_\mathrm{X}1_\mathrm{Y}\rangle + i\,\|1_\mathrm{C}0_\mathrm{X}1_\mathrm{Y}\rangle)/\sqrt{2} \to \|1_\mathrm{C}1_\mathrm{X}1_\mathrm{Y}\rangle$	$R_0(\pi/2,0)$, $S_\mathrm{X}(\pi,0)$
$(\|0_\mathrm{C}0_\mathrm{X}1_\mathrm{Y}\rangle + i\,\|1_\mathrm{C}1_\mathrm{X}1_\mathrm{Y}\rangle)/\sqrt{2} \to \|1_\mathrm{C}1_\mathrm{X}1_\mathrm{Y}\rangle$	$S_\mathrm{X}(\pi/2,-\pi/2)$
$(\|0_\mathrm{C}1_\mathrm{X}0_\mathrm{Y}\rangle + i\,\|1_\mathrm{C}1_\mathrm{X}0_\mathrm{Y}\rangle)/\sqrt{2} \to \|1_\mathrm{C}1_\mathrm{X}1_\mathrm{Y}\rangle$	$R_0(\pi/2,0)$, $S_\mathrm{Y}(\pi,0)$
$(\|0_\mathrm{C}1_\mathrm{X}0_\mathrm{Y}\rangle + i\,\|1_\mathrm{C}1_\mathrm{X}1_\mathrm{Y}\rangle)/\sqrt{2} \to \|1_\mathrm{C}1_\mathrm{X}1_\mathrm{Y}\rangle$	$S_\mathrm{Y}(\pi/2,-\pi/2)$
$(\|0_\mathrm{C}1_\mathrm{X}1_\mathrm{Y}\rangle + i\,\|1_\mathrm{C}1_\mathrm{X}1_\mathrm{Y}\rangle)/\sqrt{2} \to \|1_\mathrm{C}1_\mathrm{X}1_\mathrm{Y}\rangle$	$R_0(\pi/2,\pi)$

Supplementary Table 6. **Implementation of $|1_{\rm C}1_{\rm X}1_{\rm Y}\rangle$ measurement.** Here $|1_{\rm C}1_{\rm X}1_{\rm Y}\rangle \to |0_{\rm C}\rangle$ stands for a operation that transforms only $|1_{\rm C}1_{\rm X}1_{\rm Y}\rangle$ to $|0_{\rm C}\rangle$, and all other 7 base states to $|1_{\rm C}\rangle$.

Operation	Sequence
$P_{\rm X}(\phi)$	$R_{\rm X}(\pi/2,\phi)$, $R_{\rm X}(\pi/\sqrt{2},\phi+\pi/2)$, $R_{\rm X}(\pi/2,\phi)$
$P_{\rm Y}(\phi)$	$R_{\rm Y}(\pi/2,\phi)$, $R_{\rm Y}(\pi/\sqrt{2},\phi+\pi/2)$, $R_{\rm Y}(\pi/2,\phi)$
$|1_{\rm C}1_{\rm X}1_{\rm Y}\rangle \to |0_{\rm C}\rangle$	$P_{\rm X}(0)$, $R_0(\pi,-\pi/2)$, $P_{\rm Y}(0)$

can obtain

$$\begin{aligned} & |(\langle l| + \langle k|)\, F(0)\, |\psi\rangle|^2 - \\ & \left(|\langle l|\, F(0)\, |\psi\rangle|^2 + |\langle k|\, F(0)\, |\psi\rangle|^2\right) \\ = \ & \langle l|\, F(0)\, |\psi\rangle \langle\psi|\, F(0)\, |k\rangle + \text{h.c.} \end{aligned} \tag{26}$$

$$\begin{aligned} & |(\langle l| - i\,\langle k|)\, F(0)\, |\psi\rangle|^2 - \\ & \left(|\langle l|\, F(0)\, |\psi\rangle|^2 + |\langle k|\, F(0)\, |\psi\rangle|^2\right) \\ = \ & i\,\langle l|\, F(0)\, |\psi\rangle \langle\psi|\, F(0)\, |k\rangle - \text{h.c.} \end{aligned} \tag{27}$$

Supposing $\langle l|\, F(0)\, |m\rangle$ and $\langle k|\, F(0)\, |q\rangle$ are matrix elements that satisfy $|\,\langle l|\, F(0)\, |m\rangle\,| = |\,\langle k|\, F(0)\, |q\rangle\,| = 1$ in theory, by letting $|\psi\rangle = (|m\rangle + e^{i\phi}\,|q\rangle)/\sqrt{2}$, we have

$$\arg\frac{\langle k|\, F(0)\, |q\rangle}{\langle l|\, F(0)\, |m\rangle} \approx \arg\frac{\langle k|\, F(0)\, |\psi\rangle}{\langle l|\, F(0)\, |\psi\rangle} = \arctan\frac{(27)}{(26)}. \tag{28}$$

In practice, we let $|\psi\rangle$ traverse $(|m\rangle+|q\rangle)/\sqrt{2}$ and $(|m\rangle+ i\,|q\rangle)/\sqrt{2}$, and average their results of Supplementary Eq. (28). We define the phase of $\langle 0_{\rm C}0_{\rm X}0_{\rm Y}|\, F(0)\, |0_{\rm C}0_{\rm X}0_{\rm Y}\rangle$ to be 0, and measure the relative phases between $\langle 0_{\rm C}0_{\rm X}0_{\rm Y}|\, F(0)\, |0_{\rm C}0_{\rm X}0_{\rm Y}\rangle$ and $\langle 1_{\rm C}0_{\rm X}0_{\rm Y}|\, F(0)\, |1_{\rm C}0_{\rm X}0_{\rm Y}\rangle$, $\langle 0_{\rm C}0_{\rm X}0_{\rm Y}|\, F(0)\, |0_{\rm C}0_{\rm X}0_{\rm Y}\rangle$ and $\langle 1_{\rm C}0_{\rm X}1_{\rm Y}|\, F(0)\, |1_{\rm C}1_{\rm X}0_{\rm Y}\rangle$, $\langle 0_{\rm C}0_{\rm X}0_{\rm Y}|\, F(0)\, |0_{\rm C}0_{\rm X}0_{\rm Y}\rangle$ and $\langle 1_{\rm C}1_{\rm X}0_{\rm Y}|\, F(0)\, |1_{\rm C}0_{\rm X}1_{\rm Y}\rangle$, $\langle 0_{\rm C}0_{\rm X}1_{\rm Y}|\, F(0)\, |0_{\rm C}0_{\rm X}1_{\rm Y}\rangle$ and $\langle 1_{\rm C}0_{\rm X}1_{\rm Y}|\, F(0)\, |1_{\rm C}1_{\rm X}0_{\rm Y}\rangle$, $\langle 0_{\rm C}0_{\rm X}1_{\rm Y}|\, F(0)\, |0_{\rm C}0_{\rm X}1_{\rm Y}\rangle$ and $\langle 1_{\rm C}1_{\rm X}1_{\rm Y}|\, F(0)\, |1_{\rm C}1_{\rm X}1_{\rm Y}\rangle$, $\langle 0_{\rm C}1_{\rm X}0_{\rm Y}|\, F(0)\, |0_{\rm C}1_{\rm X}0_{\rm Y}\rangle$ and $\langle 1_{\rm C}1_{\rm X}0_{\rm Y}|\, F(0)\, |1_{\rm C}0_{\rm X}1_{\rm Y}\rangle$, $\langle 0_{\rm C}1_{\rm X}0_{\rm Y}|\, F(0)\, |0_{\rm C}1_{\rm X}0_{\rm Y}\rangle$ and $\langle 1_{\rm C}1_{\rm X}1_{\rm Y}|\, F(0)\, |1_{\rm C}1_{\rm X}1_{\rm Y}\rangle$, $\langle 0_{\rm C}1_{\rm X}1_{\rm Y}|\, F(0)\, |0_{\rm C}1_{\rm X}1_{\rm Y}\rangle$ and $\langle 1_{\rm C}1_{\rm X}1_{\rm Y}|\, F(0)\, |1_{\rm C}1_{\rm X}1_{\rm Y}\rangle$. Thus we have the phases of all 8 major elements.
